# Optimal Light Intensity for Lettuce Growth, Quality, and Photosynthesis in Plant Factories

**DOI:** 10.3390/plants13182616

**Published:** 2024-09-19

**Authors:** Mengdi Dai, Xiangfeng Tan, Ziran Ye, Jianjie Ren, Xuting Chen, Dedong Kong

**Affiliations:** 1 Institute of Digital Agriculture, Zhejiang Academy of Agricultural Sciences, Hangzhou 310021, China; daimd@zaas.ac.cn (M.D.); tanxf@zaas.ac.cn (X.T.); yezr@zaas.ac.cn (Z.Y.); nar_c2004@163.com (X.C.); 2 Shangyu Agricultural Technology Extension Center, Shaoxing 312300, China; renjianjie@icloud.com

**Keywords:** plant phenotype, plant physiology, photosynthesis, LED light

## Abstract

In agriculture, one of the most crucial elements for sustained plant production is light. Artificial lighting can meet the specific light requirements of various plants. However, it is a challenge to find optimal lighting schemes that can facilitate a balance of plant growth and nutritional qualities. In this study, we experimented with the light intensity required for plant growth and nutrient elements. We designed three light intensity treatments, 180 μmol m^−2^ s^−1^ (L1), 210 μmol m^−2^ s^−1^ (L2), and 240 μmol m^−2^ s^−1^ (L3), to investigate the effect of light intensity on lettuce growth and quality. It can be clearly seen from the radar charts that L2 significantly affected the plant height, fresh weight, dry weight, and leaf area. L3 mainly affected the canopy diameter and root shoot ratio. The effect of L1 on lettuce phenotype was not significant compared with that of the others. The total soluble sugar, vitamin C, nitrate, and free amino acid in lettuce showed more significant increases under the L2 treatment than under the other treatments. In addition, the transpiration rate and stomatal conductance were opposite to each other. The comprehensive evaluation of the membership function value method and heatmap analysis showed that lettuce had the highest membership function value in L2 light intensity conditions, indicating that the lettuce grown under this light intensity could obtain higher yield and better quality. This study provides a new insight into finding the best environmental factors to balance plant nutrition and growth.

## 1. Introduction

Lettuce (*Lactuca sativa* L.), as a one-year herbaceous plant, has become one of the main leafy vegetables because of its short growth cycle, rich variety, good quality, and its lesser tendency to be affected by pests and diseases. At present, lettuce is one of the largest hydroponic vegetables in plant factories around the world, and it is also a high-yield vegetable that can achieve stable and planned production in plant factories throughout the year. Nevertheless, as quality of life improves, so do the consumer’s preferences for high-quality vegetables. Hence, it is necessary to consider this vegetable from a comprehensive standpoint [1], taking into account the organic requirements, technological possibilities, consumer preferences, and economic factors of lettuce in order to attain top-notch vegetable cultivation and ensure the economic sustainability of plant factories [2].

Artificial lighting is a crucial factor for plant cultivation in indoor farming. A simulation study on lettuce found that vertical factories could enhance the utilization of land, water, and nutrients compared to traditional greenhouses in Sweden, the Netherlands, or the United Arab Emirates. However, this improvement comes at the expense of higher energy requirements due to the use of artificial lighting [2]. The growth and quality of plants are significantly impacted by artificial lighting. Plants are not sensitive to all light, but they are remarkably selective. Different plants are influenced by varying light quality, intensity, and photoperiods [3,4]. For example, blue light at 430–450 nm promotes germination, while red light at 640–660 nm promotes photosynthesis and flowering [5]. In a long-term study of the effects of light regime on the growth and quality of lettuce, it was found that the cultivar of lettuce determined the optimal light quality and intensity for growth [6]. And excessive light intensity (300 μmol m^−2^ s^−1^) caused tipburn [7]. In addition, frequent shifts of light/dark cycles (8 h of light and 4 h of darkness) promoted lettuce growth and the partial quality index (soluble sugar and soluble protein) [8]. According to Pennisi et al., a ratio of red to blue light of 3 had the best indoor sustainable cultivation effect on lettuce [9]. Understanding the effect of different light properties on lettuce growth will be helpful in the development and application of LED light sources in plant factories.

In plant cultivation, the modulation of light intensity is a means of optimizing biomass productivity and the synthesis of specific compounds [10]. The amount of light intensity has a significant impact on the leaf area, accumulated dry mass, and total phenol content [11,12]. Pennisi et al. found that 250 μmol m^−2^ s^−1^ was the ideal light intensity for basil development in a controlled environment, with an increase in chlorophyll content and fresh and dry weight [13]. The effects on lettuce grown in varying red–blue LED light intensities of 150, 250, and 350 μmol m^−2^ s^−1^ were investigated. Based on fluoride-resistant acid phosphatase (FRAP) and 2,2-diphenyl-1-picrylhydrazyl (DPPH) studies, the maximum concentrations of anthocyanins, polyphenols, flavonoids, and antioxidant activity were found under 350 μmol m^−2^ s^−1^ [14]. In addition, lettuce grown under a higher light intensity (250 µmol m^−2^ s^−1^ PPFD) showed changes in antioxidant activity and morphological parameters [15].

However, in order to grow high-yield and nutritious plants without wasting energy and saving investment, it is important to find a balance between plant growth and nutrition. In our study, we measured the phenotypic, physiological, and photosynthetic indices under three different light intensities (180 μmol m^−2^ s^−1^, 210 μmol m^−2^ s^−1^, 240 μmol m^−2^ s^−1^) to reveal changes in lettuce growth and development. The membership function was used to evaluate the growth adaptability of lettuce, and the best light intensity to balance the growth and quality of lettuce was found.

## 2. Results

### 2.1. Effects of Different LED Light Intensities on the Growth of Lettuce

**Figure 1:**
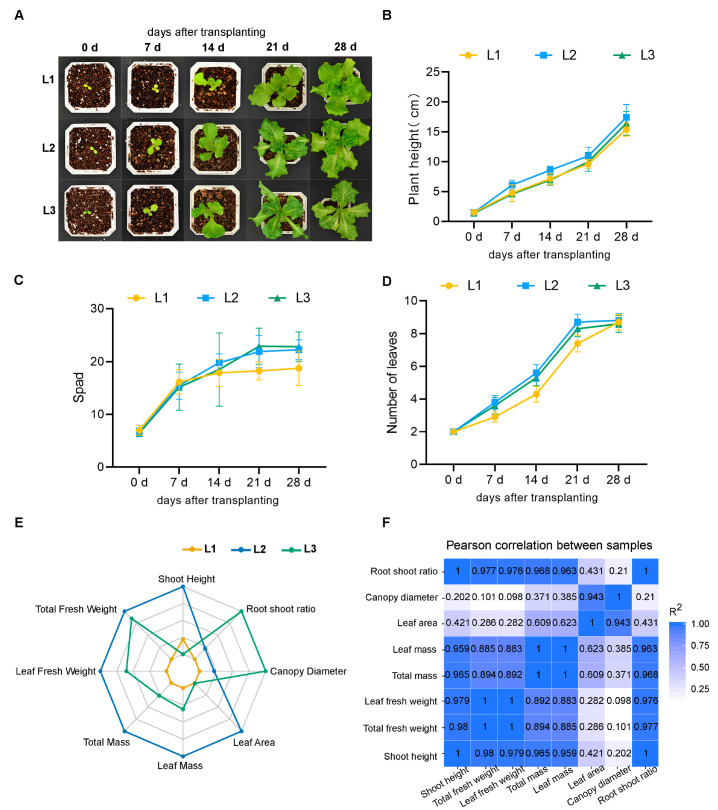
The phenotype (**A**), plant height (**B**), SPAD (**C**), and leaf number of lettuce (**D**) under different light intensities for 28 days (*p* < 0.05). (**E**) The radar chart of plant height, total/leaf fresh weight, total/leaf mass, root shoot ratio, canopy diameter, and leaf area under different light intensities. (**F**) Correlation coefficients of the main phenotypic indicators of lettuce under different light intensities (*p* < 0.05). L1, 180 μmol m^−2^ s^−1^; L2, 210 μmol m^−2^ s^−1^ ; L3, 240 μmol m^−2^ s^−1^.

To detect the effect of different LED light intensities on the growth of lettuce, we measured the phenotype of lettuce under different light intensities. Through dynamic observation of the phenotype of lettuce during 28 days of transplanting, it was found that with the increase in time, the lettuce plant height, SPAD value, and leaf number increased under the three light intensity treatments. Lettuce under the L2 treatment grew faster compared to that under the other two treatments (Figure 1A–D). In addition, we also measured other phenotypic indicators at the time of harvest. By radar chart analysis, it was found that L2 significantly affected the plant height, fresh weight, dry weight, and leaf area. However, L3 mainly affected the canopy diameter and root shoot ratio. The effect of L1 on lettuce phenotype was not significant compared with that of the others. L2 might be the appropriate light intensity for lettuce growth. Through the radar map, it can be seen that L2 is very close to the two points “canopy diameter” and “root shoot ratio”, indicating that there was an obvious effect of L2 on the canopy and root shoot ratio (Figure 1E). The correlation analysis showed that there were strong correlations among plant height, fresh weight, dry weight, and root shoot ratio. The leaf area was closely related to the maximum canopy diameter (Figure 1F).

### 2.2. Effects of Different LED Light Intensities on the Quality of Lettuce

**Figure 2:**
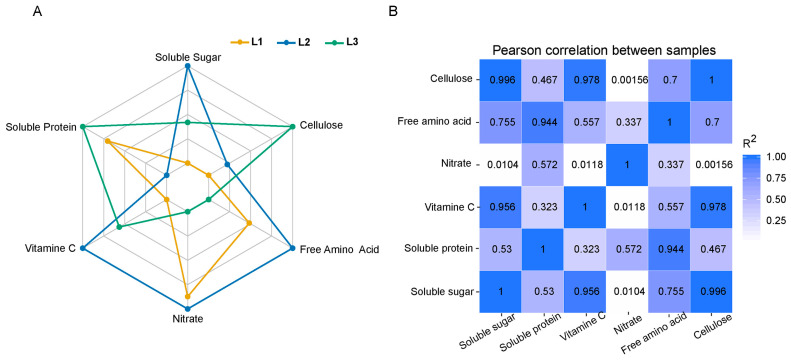
(**A**) The radar chart of total soluble sugar, soluble protein, vitamin c, nitrate, cellulose, and free amino acid under different light intensities. L1, 180 μmol m^−2^ s^−1^; L2, 210 μmol m^−2^ s^−1^ ; L3, 240 μmol m^−2^ s^−1^. (**B**) Correlation coefficients of main quality elements of lettuce under different light intensities (*p* < 0.05).

In order to explore the effects of different LED light intensities on the quality of lettuce, the contents of total soluble sugar, soluble protein, vitamin C, nitrate, and free amino acid of lettuce were determined. It was found that L2 significantly affected total soluble sugar, vitamin C, nitrate, and free amino acid via a radar chart. However, L3 mainly affected soluble protein and cellulose. The effect of L1 on lettuce quality was not significant (Figure 2A and Figure S1). The correlation analysis showed that there were strong correlations between vitamin C and soluble sugar, soluble protein and free amino acid, and cellulose and soluble sugar (Figure 2B).

### 2.3. Effects of Different LED Light Intensities on the Photosynthetic and Transpiration Characteristics of Lettuce

**Figure 3:**
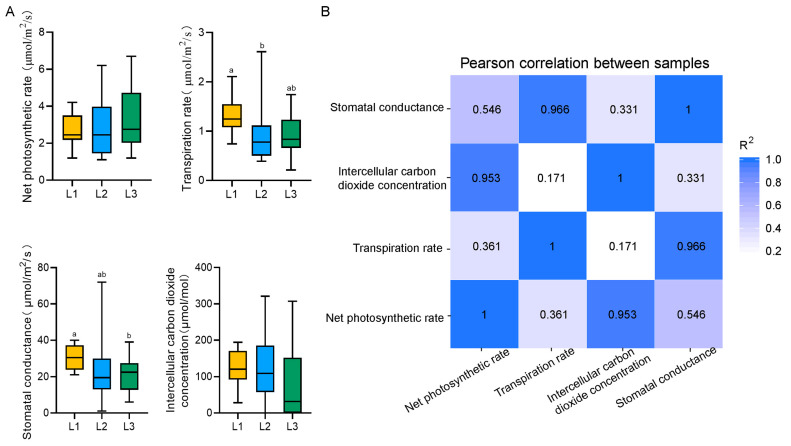
(**A**) The net photosynthetic rate, transpiration rate, intercellular carbon dioxide concentration, and stomatal conductance of lettuce under different light intensities. Different letters represent significant differences (*p* < 0.05). L1, 180 μmol m^−2^ s^−1^; L2, 210 μmol m^−2^ s^−1^; L3, 240 μmol m^−2^ s^−1^. (**B**) Correlation coefficients of photosynthetic and transpiration characters under different light intensities (*p* < 0.05).

By detecting the effect of LED light intensity on the photosynthetic and transpiration characteristics of lettuce, we found that the higher the light intensity, the lower the transpiration rate and stomatal conductance of plants. In addition, the effects of light intensity on net photosynthetic rate and intercellular carbon dioxide concentration were not significant (Figure 3A). By correlation analysis, we found that the net photosynthetic rate was closely related to the intercellular carbon dioxide concentration. In addition, stomatal conductance showed high connectivity with transpiration rate (Figure 3B).

### 2.4. Heatmap Analysis of Lettuce Character Index under Different Light Intensities

**Figure 4:**
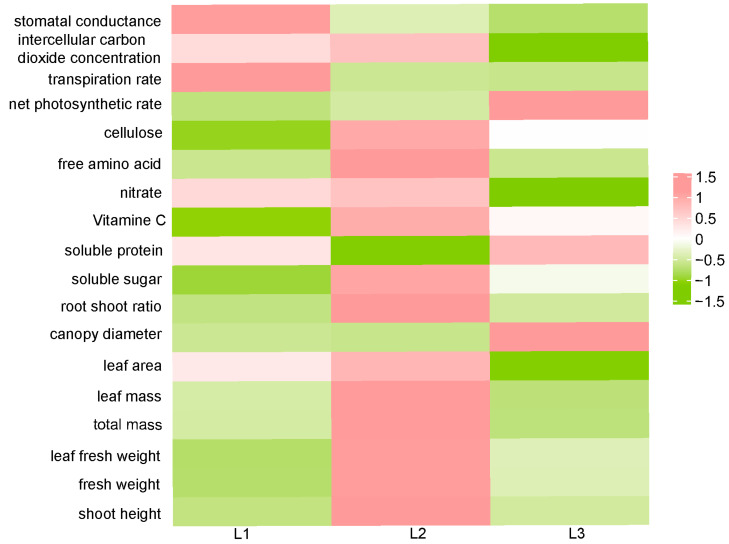
Heatmap of lettuce character indices treated with different light intensities (*p* < 0.05). L1, 180 μmol m^−2^ s^−1^; L2, 210 μmol m^−2^ s^−1^ ; L3, 240 μmol m^−2^ s^−1^.

All character indices measured in the experiment were analyzed by heatmap analysis. From the results, it was found that under L1 light intensity, four character indices were slightly up-regulated. And two indices were significantly up-regulated, including transpiration rate and stomatal conductance. Most character indices were down-regulated. Under L2 conditions, most of the character indices were significantly up-regulated, except for net photosynthetic rate, canopy diameter, transpiration rate, stomatal conductance, and soluble protein content. Lettuce grown under L3 conditions had three indices significantly up-regulated, including canopy diameter, net photosynthetic rate, and soluble protein, while all other indices were down-regulated (Figure 4).

### 2.5. Comprehensive Evaluation of Lettuce by Membership Function Values

According to Formula (1), the various character indices of different light intensities were combined into three comprehensive indicators, including phenotype index, quality index, and photosynthetic index. The standardized values of each comprehensive indicator calculated are shown in Table 1, which were used as important data for the growth adaptability evaluation of lettuce.

According to the evaluation results of different light intensities, the total membership values of three different light intensities were 0.33, 0.81, and 0.16, respectively, and the ranking of stability evaluation was L2 > L3 > L1 (Table 2). The larger the membership value, the stronger the growth adaptability of different light intensities. According to the standardized total membership function value analysis of light adaptability, the membership values of L2 and L3 were above 0.30, indicating that under the L2 and L3 light intensities, lettuce had a certain level of light growth adaptability. On the contrary, the light intensity of L1 was not suitable for the growth of lettuce. In addition, the light intensity membership value of lettuce was the highest in L2, indicating that lettuce had the strongest growth adaptability.

## 3. Discussion

Plant quality and yield, as well as photosynthesis, are intricately impacted by light factors [16,17]. This highlights how crucial it is to optimize lighting when growing plants. Plant production and growth are negatively impacted by low light intensity because of the effects on gas exchange. Conversely, excessive light might be harmful to the photosynthetic organ. Numerous studies have demonstrated that light intensity has a major impact on lettuce growth and development, plant morphology, antioxidant activity, and metabolism [7,15,18]. In this study, plant height, SPAD, and number of leaves did not increase with the increase in light intensity but reached the maximum at L2 (Figure 1B–D). In addition, we also compared the effects of different light intensities on other phenotypic indices. The results inferred that L2 significantly affected the fresh weight, dry weight, and leaf area. L3 mainly affected the canopy diameter and root shoot ratio. L1 had no discernible influence on the phenotypic qualities of lettuce (Figure 1E). This phenomenon has also been confirmed in tomatoes [19], indicating that appropriate light intensity could effectively promote the growth of lettuce. Developing adequate lighting schemes that meet the specific needs of plants is crucial to achieving optimal yields and quality of various crop cultivars. This will increase plant productivity while consuming less energy.

The physicochemical properties of plants can reflect the material exchange and metabolism process in the plant. And metabolism is closely related to the growth and development of organisms. In our study, it was found that L2 significantly affected the content of total soluble sugar, vitamin C, nitrate, and free amino acid (Figure 2A), which may be partly due to the increased carbon and nitrogen assimilation capacity of lettuce [20,21]. Compared with lettuce grown under L1 and L3, lettuce grown under the L2 treatment could better utilize carbon dioxide and nitrogen sources in the soil and convert them into sugars, nitrates, and free amino acids, respectively. And this conversion was reflected in plant yields. The lettuce grown under L2 underwent increases in fresh weight and dry weight (Figure 1E). In addition, lettuce grown under L3 had a higher content of soluble protein and cellulose compared with other treatments (Figure 2A). The increase and accumulation of soluble protein could improve the water retention capacity of plant cells. Cellulose is very hydrophilic and can absorb a lot of water [22]. This may be due to higher light intensity leading to increased water loss in plants [23]. In addition, the analysis of photosynthetic indices also found that stomatal conductance and transpiration rate decreased with the increase in light intensity (Figure 3A), indicating that under high light intensity, lettuce improved its water retention ability by reducing the transpiration rate and increasing soluble protein. SPAD reflects the green degree of leaves and indirectly represents the relative content of chlorophyll [24]. By measuring the SPAD value, we found that there was no significant difference between L2 and L3 (Figure 1C), and this phenomenon was also reflected in the measurement of net photosynthetic rate (Figure 3A). The light energy absorbed by plants is limited, and if the excessive light energy absorbed by the photosynthetic apparatus cannot be dissipated quickly, the photosynthetic efficiency will be reduced, resulting in light suppression and potential damage to the photosynthetic reaction center [25]. Moreover, the difference in fresh weight in L2 might also have been caused by the increase in photosynthetic area due to the increase in leaf area (Figure 1E).

In plant factories, finding optimal lighting schemes that can facilitate a balance of plant growth and nutritional qualities is very critical and unignorable. In the pursuit of saving energy and promoting growth, it is easy to ignore the nutritional quality of plants. Agricultural products are a dynamic composition of their physicochemical properties and changing consumer perceptions, which include sensory, nutritional, and bioactive ingredients [26]. In this study, the quality index, growth index, and photosynthetic index of lettuce were comprehensively analyzed by membership function and heatmap analysis, which were also applied to oil tea and dandelion [27,28]. It was found that under L2 conditions, most of the character indices were significantly up-regulated (Figure 4). In addition, the light intensity membership value of lettuce was the highest in L2, indicating that lettuce had the strongest growth adaptability (Table 2).

## 4. Materials and Methods

### 4.1. Plant Material and Growth Condition

Roman lettuce seeds, “Ideal-205”, were purchased from Ideal Agriculture Technology (Nanjing, China), which is a conventional variety grown in Zhejiang Province. The experiment was conducted from 5 January 2023 to 9 October 2023 in the artificial climate chamber of Zhejiang Academy of Agricultural Sciences (113.92° longitude, 27.55° latitude). Lettuce seeds were sown in a small pot (7 cm in diameter and 5 cm in height), which was filled with substrate soil (peat/vermiculite/perlite = 3:1:1). After the second true leaf of the lettuce was fully unfolded, each seedling was individually transplanted into a pot (7 cm diameter and 5 cm height) containing substrate soil and placed in an artificial climate chamber. And there were no additional nutrients provided throughout the experiment. The chamber measured 1130 mm (length) × 795 mm (width) × 1920 mm (height), and the climate chamber consisted of three microclimate chambers equipped with a control system for temperature, relative humidity, and LED lighting.

### 4.2. Treatment Design

The lettuce seedlings were cultivated in an air temperature of 25 ± 2 °C in the light and 22 ± 2 °C in the dark. The humidity was set to 85%, and the photoperiod was 16 h of light and 8 h of darkness. The three light intensity treatments were (1) 180 μmol m^−2^ s^−1^ (L1), (2) 210 μmol m^−2^ s^−1^ (L2), and (3) 240 μmol m^−2^ s^−1^ (L3). LED light sources were purchased from China Ningbo Kesheng Experimental Instrument Co., Ltd. A spectral radiometer (PLA-30, Everfine Optoelectronic Information Co., Ltd., Hangzhou, China) was used to measure the photon flux, and the correlated color temperature of the cold white LED light was about 5000 K (Figure S3). The experiment was repeated three times for each treatment with 18 plants. During the 28 d transplanting period, we observed the dynamics of lettuce plant height, leaf number, and SPAD every 7 d. After 30 d of transplanting, different treatments of lettuce were selected, and other phenotypes and physiological, photosynthetic, and transpiration indexes were determined. The remaining samples were frozen in liquid nitrogen and stored in a refrigerator at −80 °C.

### 4.3. Phenotype Measurement

The height and maximum canopy diameter of lettuce were measured by a straight ruler. The plant height is the linear distance between the highest point of the canopy leaf and the substrate soil. The maximum canopy diameter is the maximum linear distance between the canopy leaf tips. The fresh weight of the aboveground part, fresh weight of the underground part, dry weight of the aboveground part, and dry weight of the underground part were measured using a 0.0001 g analytical balance (XPR226DR/AC, Mettler Toledo, Zurich, Switzerland). Before the dry weight was determined, the sample was placed in an oven (DHG-9240A, Yiheng Co., Ltd, Shanghai, China ) at 105 °C for 15 min, and then dried at 60 °C to constant weight. A Nikon Z5 camera was used to take a picture of the leaf, and the Opencv-python 3.4.1.15 library was used to extract the leaf area of the plant. The root shoot ratio were calculated as follows:

$$\mathrm{Root}\;\mathrm{shoot}\;\mathrm{ratio}=\frac{fresh\;weight\;of\;underground\;part}{fresh\;weight\;of\;above-ground\;part}$$

### 4.4. Physiological Index Detection

For analysis, 3 healthy and fully developed lettuces were randomly selected from each treatment. Each physiological index was repeated 3 times.

#### 4.4.1. Detection of Total Soluble Sugar

The content of total soluble sugar in plants was determined by the anthrone method [29]. After removing the outer layer of fresh plant leaves, 0.3 g of leaves was ground into a powder using liquid nitrogen. The powder was combined with 10 mL of ddH_2_O in a 15 mL centrifuge tube. To extract the liquid, the mixture was then boiled for 30 min in a boiling water bath. Following that, the liquid was poured into a 25 mL volumeter bottle. At a wavelength of 630 nm, absorbance was measured, and the soluble sugar concentration was calculated using the established standard curve.

#### 4.4.2. Detection of Soluble Protein

The content of plant soluble protein was determined by Coomassie brilliant blue colorimetry [30]. Liquid nitrogen was used to grind 1.0 g of fresh plant leaves, which was then mixed with 2 mL of ddH_2_O. The supernatant was removed after 0.5–1 h at ambient temperature (20–25 °C). After adding 0.6 mL of extraction, 5 mL of Coomassie brilliant blue solution was added. After shaking, the mixture was left for two minutes. The absorbance was then measured at 595 nm, and the standard curve was used to calculate the amount of soluble protein.

#### 4.4.3. Detection of Plant Cellulose

Anthrone sulfate colorimetry was used to detect plant cellulose [31]. First, 0.2 g of plant leaves was ground and added into 60 mL of 60% sulfuric acid. The volume of the mixture was then adjusted with 60% sulfuric acid to 100 mL after 30 min. After filtration, 5 mL of supernatant was taken into a 100 mL volumetric bottle, and distilled water was added in a cold bath to adjust the volume to 100 mL. Then, 2 mL of solution was taken and 0.5 mL of 2% anthrone and 5 mL of sulfuric acid were added; it was shaken well and left for 12 min. The absorbance of the mixture was measured at 620 nm.

#### 4.4.4. Detection of Plants Nitrate

The content of nitrate in plants was measured by ultraviolet spectrophotometry [32]. First, 2.0 g of fresh plant leaves was ground into powder in liquid nitrogen and added into 20 mL of water and 1 mL of ammonia buffer, and then shaken for 30 min. The homogenate was transferred into a 50 mL volumetric bottle, 0.4 mL of 150 g/L potassium ferrocyanide solution was added, and then 0.4 mL of 300 g/L zinc sulfate solution was added. The measurement of absorbance was conducted at a wavelength of 219 nm based on the established standard curve.

#### 4.4.5. Detection of Total Free Amino Acids

The content of total free amino acids in plants was determined by the ninhydrin chromogenic method [33]. Liquid nitrogen was used to powder 0.5 g of plant leaves, which was subsequently mixed with 5 mL of 10% acetic acid. The mixture was then centrifuged, and 2 mL of the supernatant was taken out. The supernatant was added into acetate buffer (pH = 5.4) to 25 mL. Then, 2 mL of the sample extract was transferred into a 15 mL test tube and placed in a boiling water bath for 15 min. Then, 3 mL of ninhydrin solution was added to the sample dilution, and the absorbance was measured at 580 nm. The standard curve was then used to calculate the free amino acid content.

#### 4.4.6. Detection of Vitamin C

First, 2 g of fresh leaves was ground into a homogenate with 5 mL of EDTA oxalate solution. Then, it was transferred into a 25 mL volumetric bottle and added to 25 mL. Part of the homogenate was centrifuged at 3000 rpm for 10 min. Then, 1ml of supernatant was taken for determination at a wavelength of 760 nm.

#### 4.4.7. Detection of Photosynthetic and Transpiration Index

Leaf net photosynthetic rate, transpiration rate, intercellular carbon dioxide concentration, and stomatal conductance were measured by the CIRAS-2 Portable Photosynthesis System (PP Systems, New York, NY, USA) [34]. After the CO_2_ cylinder was installed, the atmospheric CO_2_ concentration was set under completely natural conditions and the lettuce leaves were placed into the probe for determination. The experiment was repeated three times for each treatment with 18 plants.

### 4.5. Evaluation and Analysis

The response of phenotype, physiology, and photosynthesis of lettuce to light intensity was inconsistent. The membership function method was used to calculate and analyze three indices (phenotype index (plant height, total/leaf fresh weight, total/leaf mass, canopy diameter, and leaf area), physiological index (total soluble sugar, soluble protein, vitamin c, nitrate, cellulose, and free amino acid) and photosynthetic index (net photosynthetic rate, transpiration rate, intercellular carbon dioxide concentration, stomatal conductance)) of lettuce under different light intensities. In order to reduce the impact of variables among different indicators on the evaluation results, the original data of each factor were standardized and converted first. The formula is as follows:

*X_ik_* = *X_jk_/X_kmax_* × 1000 (1)

*X_jk_* represents the actual measured value of the k-th index of j-th light intensity, and *X_kma_* is the maximum value of the evaluation factor in the kth index.

The membership function values of each index under different light intensities were calculated. The formula is as follows:

*U*(*X_ijk_*) = (*X_jk_* − *X_kmin_*)/(*X_kmax_* − *X_kmin_*) (2)

*U*(*X_ijk_*) is the kth evaluation factor (phenotypic index, quality index, photosynthetic index) in the jth light environment, and the value range of *U*(*X_ijk_*) is 0~1. *X_jk_* represents the value of the kth index in the jth light environment. *X_kmax_* and *X_kmin_* are the maximum and minimum values of the kth indicator among the 3 indicators.

### 4.6. Statistical Analysis

Statistical analysis was performed using GraphPad Prism 8 and SPSS 24.0 software. Significance was determined by a one-way ANOVA or independent sample *t* test (*p* < 0.05). Pearson correlation was used to analyze the correlation between the main indicators. The ggplot2 package of R was used to generate heatmaps.

## Figures and Tables

**Table 1:** Summary value of each factor of 3 evaluation indices under different light intensities. L1, 180 μmol m^−2^ s^−1^; L2, 210 μmol m^−2^ s^−1^ ; L3, 240 μmol m^−2^ s^−1^.

Treatment	Phenotypic Index	Quality Index	Photosynthetic Index
L1	769	666	937
L2	974	897	822
L3	776	767	733

**Table 2:** Standardized value of each factor of 3 evaluation indices under different light intensities. L1, 180 μmol m^−2^ s^−1^; L2, 210 μmol m^−2^ s^−1^; L3, 240 μmol m^−2^ s^−1^.

Treatment	Phenotypic Index	Quality Index	Photosynthetic Index	Total Membership Value	Rank
L1	0.00	0.00	1.00	0.33	3
L2	1.00	1.00	0.44	0.81	1
L3	0.03	0.44	0.00	0.16	2

## Data Availability

The original contributions presented in the study are included in the article/Supplementary Material, further inquiries can be directed to the corresponding authors.

## Supplementary Materials

The following supporting information can be downloaded at: https://www.mdpi.com/article/10.3390/plants13182616/s1, Figure S1: The maximum canopy diameter, leaf area, fresh weight of above ground and underground, dry weight of above ground and underground of lettuce under different light intensity.; Figure S2: The contents of total soluble sugar, soluble protein, vitamin c, nitrate, cellulose and free amino acid of lettuce under different light intensity. Figure S3: The photon flux for three scenarios measured by PLA-30.
