# Supplementary material for: Optimal Light Intensity for Lettuce Growth, Quality, and Photosynthesis in Plant Factories
Source: Plants (Basel). 2024 Sep 19;13(18):2616. doi: 10.3390/plants13182616 (PMC11434889; doi:10.3390/plants13182616)
Supplement: Supplementary file 1 [file plants-13-02616-s001.zip › plants-3111093-supplementary.pdf]

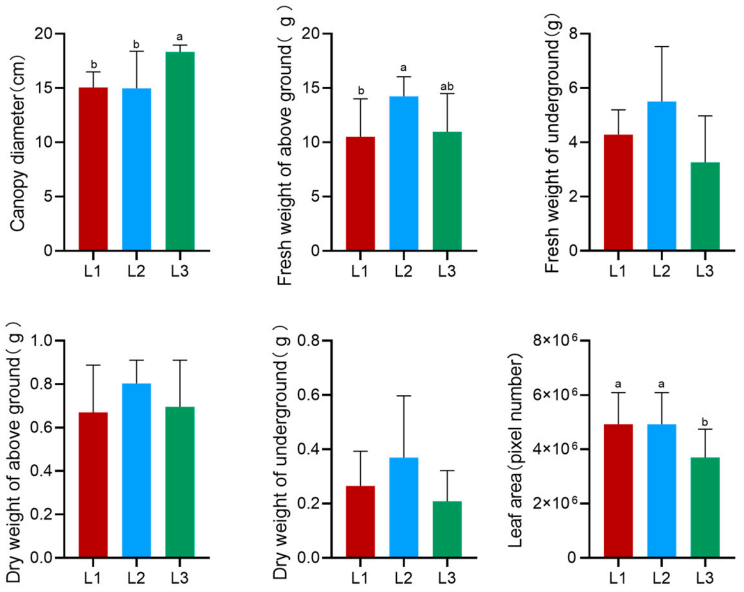

Figure S1 The maximum canopy diameter, leaf area, fresh weight of above ground and underground, dry weight of above ground and underground of lettuce under different light intensities. L1, 180 μmol m^{-2} s^{-1}; L2, 210 μmol m^{-2} s^{-1} ; L3, 240 μmol m^{-2} s^{-1}.

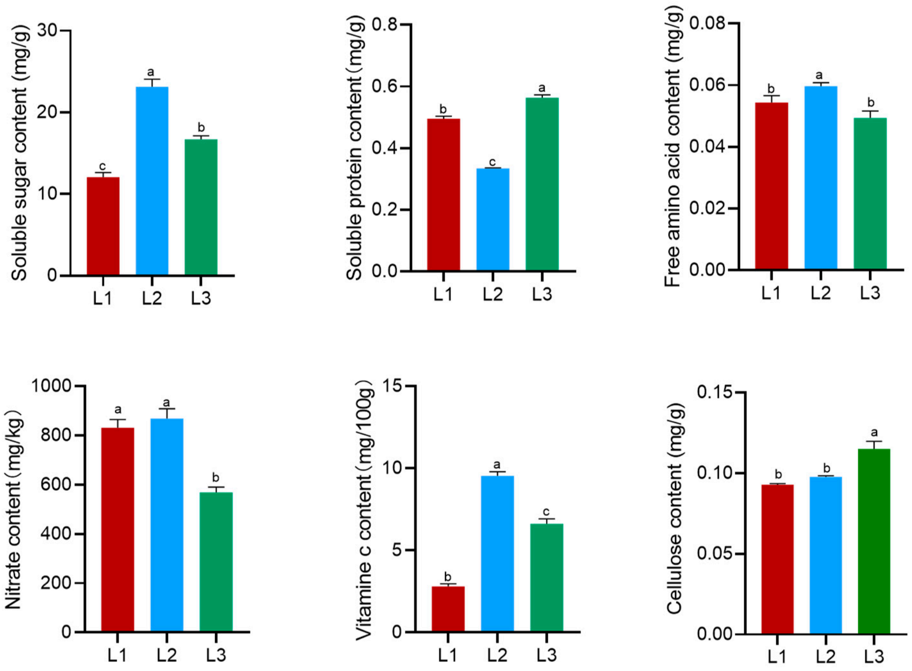

Figure S2 The contents of total soluble sugar, soluble protein, vitamin c, nitrate, cellulose and free amino acid of lettuce under different light intensities. L1, 180 μmol m^{-2} s^{-1}; L2, 210 μmol m^{-2} s^{-1} ; L3, 240 μmol m^{-2} s^{-1}.

L1

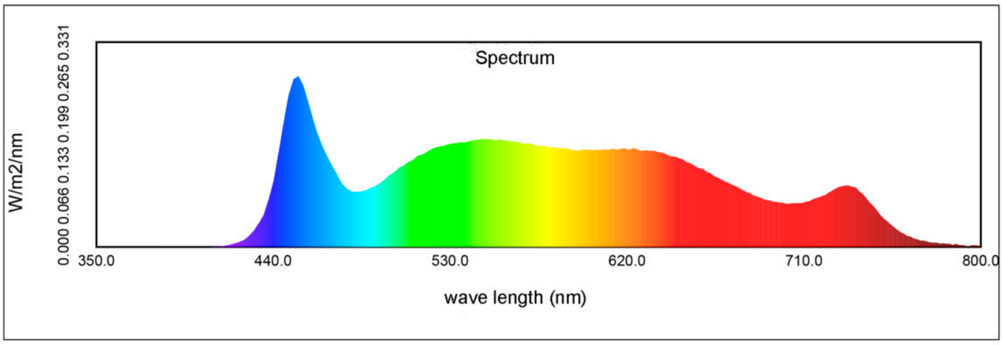

Items	value	Items	value	Items	value
PPFD(umol/m²/s)	180.42	Ev(lx)	11568.98	Ee(W/m)	4.3317E1

L2

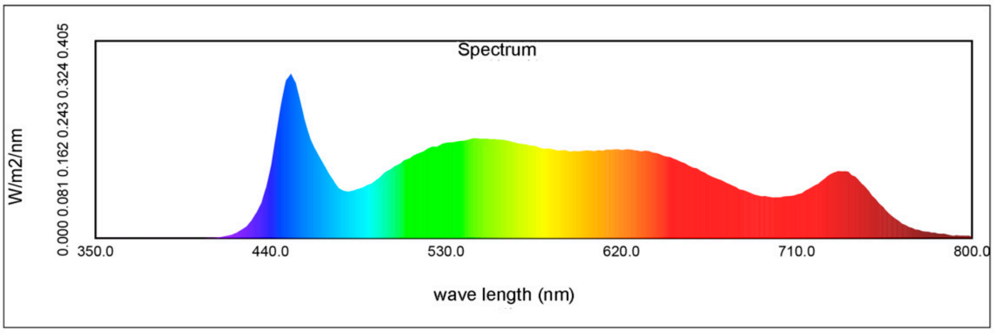

Items	value	Items	value	Items	value
PPFD(umol/m²/s)	207.87	Ev(lx)	13385.64	Ee(W/m)	5.1254E1

L3

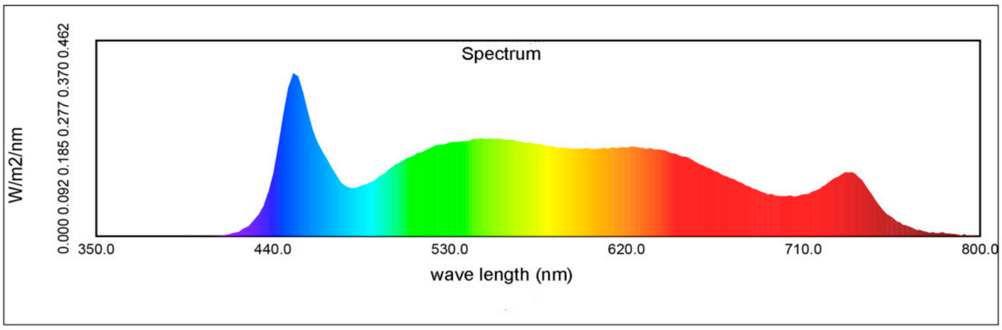

Items	value	Items	value	Items	value
PPFD(umol/m²/s)	239.80	Ev(lx)	15331.19	Ee(W/m)	5.7974E1

Figure S3 The photon flux for three scenarios measured by PLA-30. The "value" in the table represents the light intensities. L1, 180 μmol m^{-2} s^{-1}; L2, 210 μmol m^{-2} s^{-1}; L3, 240 μmol m^{-2} s^{-1}.
